# Sequence and structural variation in the genome of the *Biomphalaria glabrata* embryonic (Bge) cell line

**DOI:** 10.1186/s13071-018-3059-2

**Published:** 2018-09-04

**Authors:** Nicolas J. Wheeler, Nathalie Dinguirard, Joshua Marquez, Adrian Gonzalez, Mostafa Zamanian, Timothy P. Yoshino, Maria G. Castillo

**Affiliations:** 1 Department of Pathobiological Sciences, School of Veterinary Medicine, University of Wisconsin, Madison, WI USA; 2 Department of Biology, New Mexico State University, Las Cruces, NM USA

**Keywords:** Bge, Genome sequence, Variant calling, Karyotype, *Biomphalaria glabrata*, *Schistosoma mansoni*

## Abstract

**Background:**

The aquatic pulmonate snail *Biomphalaria glabrata* is a significant vector and laboratory host for the parasitic flatworm *Schistosoma mansoni*, an etiological agent for the neglected tropical disease schistosomiasis. Much is known regarding the host-parasite interactions of these two organisms, and the *B. glabrata* embryonic (Bge) cell line has been an invaluable resource in these studies. The *B. glabrata* BB02 genome sequence was recently released, but nothing is known of the sequence variation between this reference and the Bge cell genome, which has likely accumulated substantial genetic variation in the ~50 years since its isolation.

**Results:**

Here, we report the genome sequence of our laboratory subculture of the Bge cell line (designated Bge3), which we mapped to the *B. glabrata* BB02 reference genome. Single nucleotide variants (SNVs) were predicted and focus was given to those SNVs that are most likely to affect the structure or expression of protein-coding genes. Furthermore, we have highlighted and validated high-impact SNVs in genes that have often been studied using Bge cells as an *in vitro* model, and other genes that may have contributed to the immortalization of this cell line. We also resolved representative karyotypes for the Bge3 subculture, which revealed a mixed population exhibiting substantial aneuploidy, in line with previous reports from other Bge subcultures.

**Conclusions:**

The Bge3 genome differs from the *B. glabrata* BB02 reference genome in both sequence and structure, and these are likely to have significant biological effects. The availability of the Bge3 genome sequence, and an awareness of genomic differences with *B. glabrata*, will inform the design of experiments to understand gene function in this unique *in vitro* snail cell model. Additionally, this resource will aid in the development of new technologies and molecular approaches that promise to reveal more about this schistosomiasis-transmitting snail vector.

**Electronic supplementary material:**

The online version of this article (10.1186/s13071-018-3059-2) contains supplementary material, which is available to authorized users.

## Background

*Biomphalaria glabrata* is an aquatic pulmonate snail that serves as a vector and/or experimental intermediate host for several human-infective helminths, including the platyhelminths *Schistosoma mansoni* [1] and *Echinostoma* spp. [2] and the nematode *Angiostrongylus cantonensis* [3]. Multiple strains of this snail have been isolated that support laboratory culture of the complete life-cycle of the blood fluke *S. mansoni*. These also provide sources of genomic variation that lead to diverse phenotypic outcomes, such as differences in the strains’ susceptibility or resistance to schistosome infection [4–8]. This diversity of snail strains and their availability has provided an important resource for identifying new genetic factors that may be important to naturally occurring resistant populations.

In order to further understand the development of *Schistosoma* parasites in the molluscan intermediate host, and as part of the ongoing efforts to control the transmission of schistosomiasis to humans, the genome of *B. glabrata* (BB02 strain) was recently sequenced, assembled, and annotated [9]. Furthermore, a linkage map has been constructed to arrange many of the original genomic scaffolds to linkage groups that likely represent the 18 haploid chromosomes characteristic of *B. glabrata* [10]. These resources will enable the development and utilization of new genetic tools in this system. For instance, selection and genotyping by the sequencing of restriction site-associated DNA markers (RADseq) has recently led to the identification of a gene cluster that is hypothesized to be involved in schistosome identification and clearance [11], and reverse genetics through RNA interference (RNAi) has been used to functionally annotate numerous snail genes [12–14].

In addition to the diverse strains of *B. glabrata*, the *Biomphalaria glabrata* embryonic (Bge) cell line has also been developed for the study of this important vector. The Bge cell line was originally isolated and established from 4–5 day-old embryos of an albino *B. glabrata* snail strain that exhibited high susceptibility to *S. mansoni* infections [15], and it is the only lophotrochozoan immortalized cell line currently available. Since then, it has proved to be a valuable resource, being extensively used to study snail vector-larval fluke interactions, particularly involving *Echinostoma* spp. and *Schistosoma* spp. Bge cells in culture have the ability to support the development of the intramolluscan stages of these trematodes, enabling the design of experiments and development of techniques that were not previously possible when utilizing snails as laboratory hosts [16–18]. Additionally, Bge cells exhibit a hemocyte-like morphology and behavior, which includes the encapsulation of schistosome sporocysts [19, 20], a critical step in the natural response of resistant snails to infection [21]. In the same way that research efforts and new approaches have been enhanced by the availability of the *B. glabrata* genome sequence, the data obtained from the sequencing of the Bge cell line’s genome will provide another important tool to further facilitate efforts toward understanding the crucial relationship between this molluscan vector and schistosome parasites.

Like other immortalized cell lines, Bge is likely to contain a significant amount of genetic variation when compared to the organism from which it was originally derived [22]. Indeed, an updated karyotyping of two Bge subcultures (Bge1 and Bge2) revealed extensive chromosomal aneuploidy with modal chromosome counts of 63 and 67, respectively, instead of the original 2n of 36 [23]. These and other forms of genomic variation are likely to have unpredictable effects on Bge cell function and/or behavior. To complement the information provided by the *B. glabrata* genome, and to improve the utilization of Bge cells to study vector-parasite interactions, we have sequenced the genome of a laboratory Bge cell subculture (designated Bge3) and resolved representative karyotypes. In addition, we identified single-nucleotide variants (SNVs) between Bge3 and the *B. glabrata* snail genome and predicted functional consequences of these variants at the protein level. Predicted high-impact variants were analyzed for patterns of enrichment with respect to fundamental cell processes, and those found in selected genes of interest were verified with Sanger sequencing. These data complement the recently assembled *B. glabrata* genome and will significantly augment the utility of this cell line as a resource for the study of snail-parasite interactions. Knowledge of Bge genetic variation will allow for more careful experimental design and will make possible new technological advancements in this *in vitro* system.

## Results

### Genome quality and coverage

**Fig. 1:**
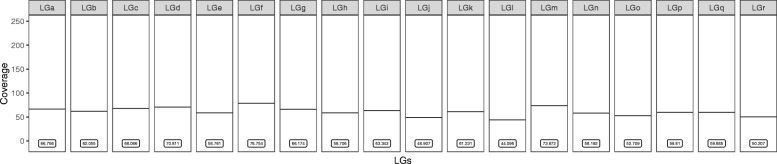
Average read depth coverage (RDC) for the Bge3 cell line genome mapped to the 18 largest *B. glabrata* linkage groups (LGs). Illumina reads from the Bge3 genome were aligned to the *B. glabrata* BB02 reference and mapped to LGs. Each point represents the average RDC for one scaffold, and only scaffolds > 10,000 base pairs are shown. Horizontal lines represent the mean RDC for the entire LG, which is displayed at the bottom of each panel

We report here the sequencing of the genome of the *Biomphalaria glabrata* embryonic cell line subculture 3 (Bge3). Illumina HiSeq sequencing yielded 331 million reads, 98.6% of which successfully mapped to the recently released *B. glabrata* strain BB02 reference genome [9]. Read depth coverage (RDC) surpassed an average of 40× for each of the largest 18 linkage groups (LGs) and ranged between 44× – 79× (Fig. 1). Additionally, the wide range of mean RDC among the 18 largest LGs alluded to possible aneuploidy in Bge3.

### SNV analysis reveals variation in important gene classes

To probe the genomic differences between Bge3 and the reference *B. glabrata* BB02 strain, we identified, filtered, and annotated Bge3 variants. Variant calling with bcftools [24] identified 12,618,003 variants. Because indels and structural variants are difficult to confidently call with short-read data [25], we removed these and retained the remaining 10,489,717 single nucleotide variants (SNVs). SNVs were filtered for quality and biallelism, and a final dataset that included 10,031,395 SNVs was retained (~11 SNVs per kilobase), reflecting the divergence of this cell line from the reference *B. glabrata* genome. For comparison, the HeLa and U87MG cell lines were found to have 3,026,053 and 2,384,470 heterozygous SNVs, or ~1 and ~0.74 SNVs per kilobase, respectively, when compared to the human reference [26, 27]. Differences in variant calling and filtering is likely to account for some of the discrepancy between the human and snail systems, as well as the contrasting qualities of the reference genomes.

**Fig. 2:**
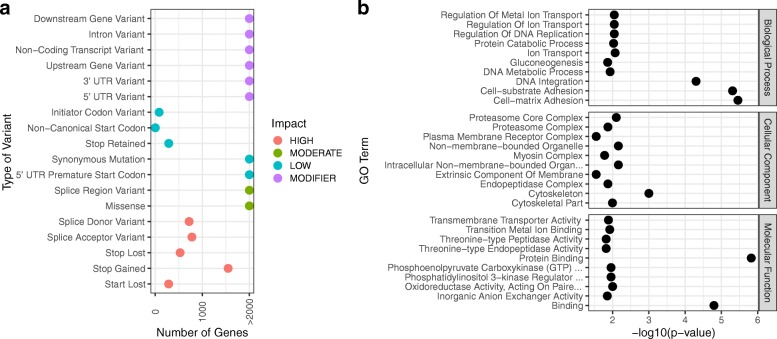
Annotation of genome-wide single-nucleotide variants (SNVs) and analysis for Gene Ontology (GO) term enrichment. **a** SNVs were filtered for quality and biallelism, and the filtered SNVs were annotated by their predicted impact on protein-coding genes. The number of variants for each classification of predicted impact is shown. **b** High-impact SNVs (a, red) were analyzed for GO term enrichment using Fisher’s exact test. The ten GO terms with lowest *p*-value in each of the three main GO classes are shown

We used SnpEff [28] to assess how these variants might affect the function of the protein products encoded by the genes containing SNVs. SnpEff incorporates gene annotations to predict whether called SNVs will have an effect on the final protein product, and it classifies these SNVs into low, moderate, or high-impact based on the type of variant effect (or modifier if the change is not predicted to have an effect). Predicted high-impact SNVs (one or more SNV that cause a change in splice acceptor/donor region, a loss of start or stop codon, or a gain of stop codon) were found in 3,277 transcripts (Fig. 2a). Interestingly, genes involved in the cytoskeleton, gluconeogenesis, and ion transport, among others, were found to be overrepresented in this highly impacted dataset (Fig. 2b).

Knowing the risk for false-positives in variant calling techniques, 151 genes of interest that were predicted to have high-impact SNVs were selected for more detailed analysis and verification with Sanger sequencing. Genes of interest were grouped into five functional categories: genes involved in immune/stress related pathways, apoptosis, transcriptional regulation, cell cycle, and epigenetic regulation. However, we found no high-impact variants in any of the genes involved in epigenetic regulation (BgDMNT1, BgDMNT2, and BgMBD2/3 [29]). Prior to Sanger sequencing, SNVs were manually confirmed to have high coverage and confidence (> 30 reads mapping to the locus with > 10 reads supporting the alternative allele) by examination of the genome alignment file, and PCR primers were designed to amplify fragments of 83 of these genes. Seventy-five fragments were successfully amplified and sequenced, and 66 (88%) of the predicted SNVs were confirmed (Additional file 1). These data confirm that genomic variation exists in many genes that are often studied in the Bge cell line, and they provide support for the variant calling and filtering pipeline used.

### Molecular karyotyping suggests a mixed aneuploid cell population

The original Bge cell line was described as having a diploid (2n) chromosomal count of 36, the same as the *B. glabrata* snail isolate from which it was derived [15, 30]. However, subsequent culture passage of Bge cells in different laboratories resulted in extensive aneuploidy in these subcultures [23]. In order to investigate the possibility of aneuploidy in the Bge3 laboratory subculture, molecular karyotyping of Bge3 cells was performed using the short-read genomic data. This approach has been extensively utilized in other eukaryotic groups including fungi and protozoans, where short genomic sequencing reads are used to estimate chromosome copy number (CCN) *in silico*, without the need to perform cytogenetic karyotyping [31–35]. Molecular karyotyping typically uses two methods to estimate the CCN. The first approach uses the stratification of the average read depth coverage (RDC) of chromosomes: more reads mapping to a given chromosome indicates a higher CCN. The second approach estimates the CCN by calculating SNV allele observation frequencies: the ratio of reads supporting the alternative allele to total reads mapped to that locus correlates to CCN [36]. Estimated CCN for each chromosome is then used to indicate whether the sample is euploid or aneuploid.

We measured RDC at each nucleotide position and averaged the RDC across each scaffold. Mean RDC ranged between 44–79× for the Bge3 genome mapped to the 18 largest LGs from the *B. glabrata* reference (Fig. 1). This variation in chromosomal RDC could imply that Bge3 is aneuploid. However, we realigned a subset of *B. glabrata* BB02 paired-end reads back to its own reference (99.93% mapping rate) and calculated RDC, and the mean RDC for each LG also varied considerably in this dataset (Additional file 2). *Biomphalaria glabrata* is diploid, so similar variation in chromosomal RDC in this sample of reads suggests that the differences in RDC in the Bge3 dataset cannot be definitively linked to CCN.

**Fig. 3:**
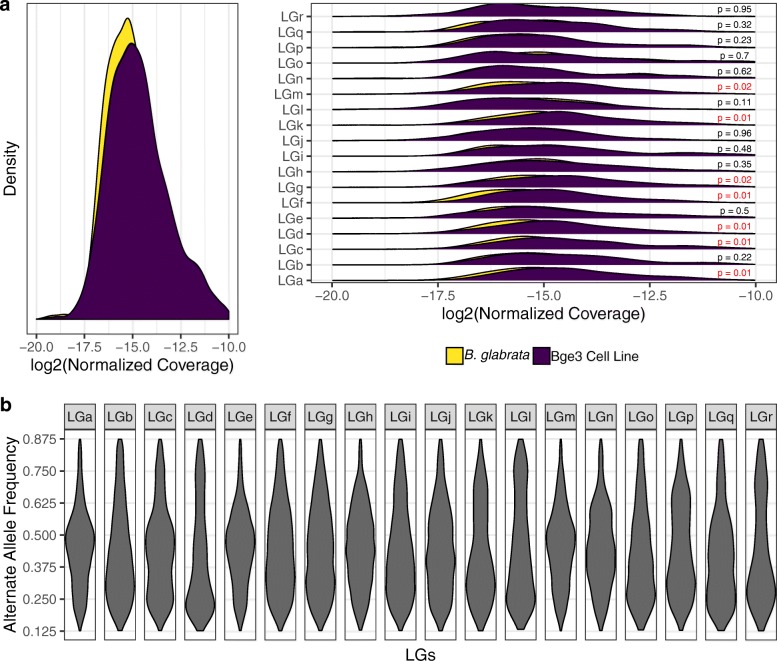
Molecular karyotyping of Bge3 cell line with read depth coverage and single-nucleotide variant allele frequency. Short paired-end Illumina reads from Bge3 and *B. glabrata* were mapped to version 1 of the strain BB02 reference genome. **a** Total normalized coverage for Bge3 and *B. glabrata* reads were similar (left), while coverage distributions for 7 of the 18 largest linkage groups (LGs) were significantly different (right, *P* < 0.05, highlighted in red). The Kolmogorov-Smirnov test was used to test the null hypothesis that the two LG RDC samples are drawn from the same distribution. **b** SNVs were filtered for quality and biallelism and grouped by LG. Reads supporting the alternative allele for each SNV were counted and plotted as a frequency to total reads at that site. Given a population with stable chromosome copy number (CCN), alternative allele frequency from independently inherited LGs should correlate to CCN. Allele frequencies greater than 0.875 and less than 0.125 were trimmed to theoretically accommodate octosomies. Frequencies in our data do not coalesce around predictable ratios, suggesting that CCN as measured by allele frequency is masked by a mixed aneuploid population

When chromosomal RDC of the Bge3 and *B. glabrata* alignments was compared, total RDC depth did not differ, yet there were significant differences between RDC distributions across some of the 18 LGs (Fig. 3a). These differences could be explained by aneuploid CCN in the Bge3 genome. However, the shifts in distributions did not appear to have discrete integer covariates, making accurate predictions of CCN impossible.

As an alternative to using average chromosomal RDC, we calculated allele observation frequencies for SNVs found in the 18 LGs. We plotted the number of observations (i.e. reads) supporting the alternative allele divided by the total number of observations at that position (Fig. 3b). Ratios varied widely for each of the 18 LGs and did not coalesce around discrete, predictable frequencies. These data suggest that the Bge3 cell line is aneuploid, and that it is a mixed non-clonal aneuploid population.

### Bge3 is a mixed, non-clonal population of highly aneuploid cells

**Fig. 4:**
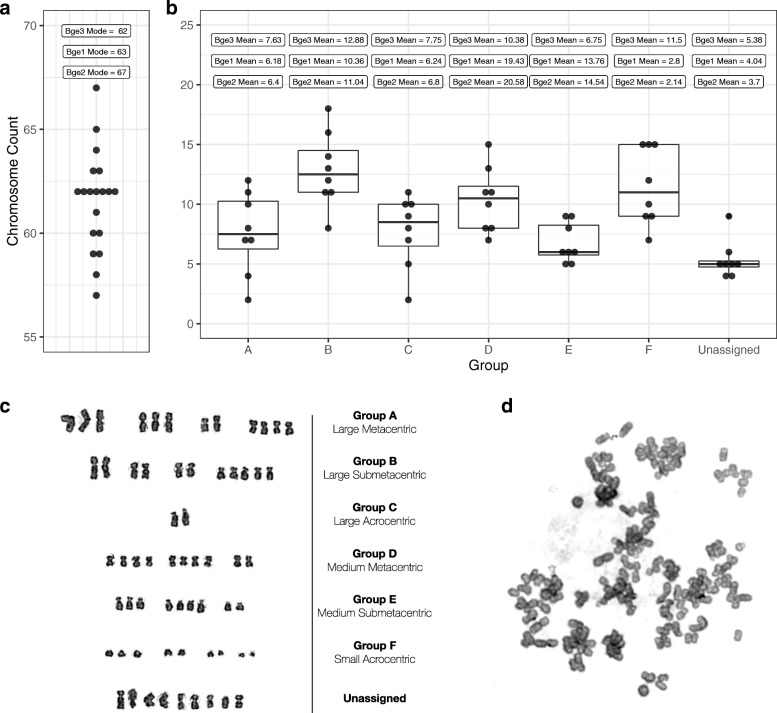
Karyotype of Bge3 cells reveals mixed aneuploidy and differences among other subcultures of Bge. **a** Chromosomes were tallied for 20 cells; each point represents a single cell. Modal counts from karyotypes of other Bge subcultures are included for comparison [20]. **b** Karyotype counts and box plots for 6 groups previously established based on chromosome size and centromere position, and one group of unassigned remainders. Group means from karyotypes of other Bge subcultures are included. **c** A representative karyogram of one cell from the Bge3 analysis. **d** An example of a putative tetraploid cell

Because short-read sequencing supported the hypothesis of aneuploidy but did not provide an estimate of CCN, a classical cytogenetic karyotyping of Bge3 was performed (Fig. 4). As predicted, we found significant intrapopulation variation in both total chromosomal count and CCN (Fig. 4a, b, Additional files 3, 4, 5, 6, 7, 8 and 9). The modal chromosomal count of 20 randomly selected cells was 62, very near the published modes of 63 and 67 for Bge1 and Bge2, respectively, and greater than the original 2n = 36 of Bge and *B. glabrata* (Fig. 4a) [23]. Interestingly, we also found that approximately 10% of the subset of cells analyzed had a far greater number of chromosomes (around 120), suggesting possible tetraploid-like subpopulations (Fig. 4d, Additional file 10). These cells were not included in modal calculations. We were unable to assign Bge3 cell chromosomes to the *B. glabrata* karyotype [30], but we were able to classify them into the 6 groups described by Odoemelam et al. [23], with some confounding chromosomes placed in an “Unassigned group” (Fig. 4b). When compared to karyotypes of other Bge subcultures, Bge3 cells exhibited similar counts for groups A, B, and C greater counts for group F and the unassigned group, but far fewer in groups D and E.

## Discussion

We report here the genome sequence of a subculture of the only immortalized lophotrochozoan cell line, the Bge3 cell line. The *B. glabrata* BB02 genome was used as a reference in order to compare Bge3’s genome with that of the organism from which it was derived. To complement the genomic sequence data, the karyotype of the laboratory Bge3 cell subculture was obtained and compared to that of the original Bge culture [15, 30], and to that of two other Bge subcultures, Bge1 and Bge2 [23].

Because there was sufficient coverage across the Bge cell genome (Fig. 1), we first attempted to measure chromosome copy number (CCN) *in silico* using read depth coverage (RDC) and single-nucleotide variant (SNV) allele observation frequencies. These techniques have been shown to be robust in clonal organisms and more established systems like yeast and protozoans, and have been used for clinical assessment of aneuploidy in preimplantation human embryos [31–37]. However, although there are apparent differences between LG coverage for Bge3 and the *B. glabrata* BB02 reference (Fig. 3a), mean RDC for LGs did not stratify as expected (Fig. 1), but instead showed normal variation that is likely due the molecular techniques used in the course of sequencing (Additional file 11). Likewise, allele observation frequencies did not coalesce around predictable ratios that correspond to CCN (Fig. 3b). For a trisomy, for instance, one would predict that the allele observation frequencies for all heterozygous SNVs on that set of homologous chromosomes would coalesce around 33% and 67%.

A nonclonal and aneuploid population of cells would have a CCN that is indiscernible to these *in silico* methods, and the results of our analyses suggested this phenomenon in the Bge3 cell line. Since previous karyotyping of other subcultures of Bge cells revealed nonclonal aneuploid cell populations, we hypothesized based on our *in silico* results that the same would hold true for the Bge3 subpopulation. Indeed, cytogenetic karyotyping confirmed this hypothesis. Interestingly, while differences in CCN exist among the 3 Bge cell subcultures tested to date, the total modal count remains remarkably similar (63 for Bge1, 67 for Bge2, and 62 for Bge3) (Fig. 4). The most striking differences in CCN were found in groups D, E and F, in which Bge3 cells contained greater chromosome numbers in group F and far less in groups D and E. This likely reflects a true difference in chromosome composition between the different Bge cell subpopulations examined to date.

The tetraploidy that we observed (Fig. 4d) is not unique to Bge3 cells but is a regularly observed phenomenon that is often caused by chromosome nondisjunction in immortalized cell lines [38]. Tetraploidy was found to occur in about 12% of N/TERT-1 cells [38], which comports well with our observations in Bge3 cells. No mention of tetraploid cells is given in previous karyotypes of Bge1 and Bge2 [23], so this may represent another distinguishing feature of the Bge3 population.

In addition to variation in chromosomal structure and number, we analyzed SNVs that were predicted to have effects at the protein level. An enrichment analysis of high-impact SNVs revealed several interesting trends. First, Bge3 contains 255 high-impact variants in genes predicted to function as a cytoskeletal component (GO:0005856, GO:0044430). It is well known that cancer and immortalized transformed cell lines like Bge often display chromosomal variations in ploidy and structure [39, 40], and much of chromosomal and mitotic homeostasis is governed by proper cytoskeletal function [41]. Indeed, cytoskeletal defects are likely to cause chromosomal instability in cancer cell lines [42], so it is not surprising to see high-impact SNVs overrepresented in cytoskeletal proteins. These may contribute to the substantial aneuploidy exhibited in the three different Bge strains that have been examined cytogenetically, and they may be linked to the process of immortalization and selection due to continuous laboratory passaging of this cell line.

Secondly, the overrepresentation of high-impact variants in enzymes involved in sugar biosynthesis (GO:0006094, GO:0019319) comports well with the expectations associated with proliferative cell lines, as they must reprogram their metabolic pathways in order to support energy requirements of immortalization [43]. Likewise, tumors undergo a similar process, and an increased reliance on glucose metabolism - the Warburg effect - is one of the hallmarks of cancer. Indeed, some cancers, like fumarate hydratase (FH)-deficient kidney cancer, are explicitly caused by loss-of-function mutations in enzymes involved in important metabolic pathways, and these tumors adjust their metabolism accordingly [44]. The immortalization of Bge cells could also have occurred as a result of similar changes. Furthermore, it has been shown that infection of *B. glabrata* by various trematodes including *S. mansoni* [45–47], *E. caproni* [48], and *E. paraensei* [49] and the nematode *Angiostrongylus cantonensis* [50] greatly modifies the snail’s glucose metabolism and storage pathways. *B. glabrata* glycogen reserves decrease after *A. cantonensi*s and *S. mansoni* infection, and a significant reduction in the concentration of free glucose in the hemolymph is also a consequence of larval infections. Given these observations and the high-impact variants found in genes involved in sugar biosynthesis, it would be interesting to see how these affect carbohydrate metabolism in Bge cells.

In addition to high-impact variants overrepresented in specific gene ontology classes, we focused on modifications in genes involved in cell cycle regulatory processes, such as apoptosis and transcriptional regulation. We found high-impact variants in many of these genes, some of which may be associated with the process of immortalization (Additional file 12).

Bge cells were originally derived from susceptible snails, and due to morphological and physiological similarities with *B. glabrata* hemocytes, the Bge cell line is often used as an *in vitro* system to study snail cell responses to trematode infection [19, 29, 51, 52]. Although this model often replicates aspects of the whole-animal *in vivo* response and is useful for extrapolating conclusions to the whole snail, it is not always clear how accurate these extrapolations may be. To address this concern, we selected genes that are associated with the production of proteins with immune-related functions, such as pattern recognition (lectins, thioester-containing proteins, macrophage mannose receptors) or stress response (antioxidants, heat shock proteins), and we manually verified the presence of the predicted SNVs in these genes. Because our results confirmed the presence of the vast majority of the selected variants, and because some of these could cause idiosyncratic behaviors in Bge-mediated parasite recognition and encapsulation, it will be necessary to consider these and other variants when designing experiments probing the immunological behavior of Bge cells.

For instance, it has been shown that H_2_O_2_ is a critical agent in hemocytes’ schistosomicidal activity and that higher expression and activity of a *B. glabrata* superoxide dismutase (SOD1), an enzyme that catalyzes the conversion of reactive oxygen species (O_2_-) to H_2_O_2_, may play a role in resistance to infection [53, 54]. Two divergent haplotypes within the terminal 3' intron of SOD1 have been shown to be linked to susceptibility or resistance [55], though these variants may not be causal and may be epistatic with other resistance effectors [56]. Interestingly, genomic reads from Bge3 that mapped to this intron all contain the C haplotype that is linked with susceptibility, which may help explain the cell line’s suitability for *in vitro* co-cultivation with *S. mansoni* intramolluscan stages.

The phosphoinositide (PI3K) pathway is also a route for H_2_O_2_ production, and it has been shown that pharmacologic inhibition of this pathway can lead to reduced H_2_O_2_ production in hemocytes [57]. We found high-impact variants in 2 genes involved in phosphatidylinositol 3-kinase regulation (GO:0035014), and it is possible that Bge may provide a useful *in vitro* knockout model for functional investigations of these genes. It is likely that other gene variants existing in Bge cells can be utilized by applying similar approaches, but it will require a careful analysis of the sequence of interest prior to experimentation.

## Conclusions

The Bge cell line was developed from the embryos of an albino strain of *B. glabrata* that were first collected in Puerto Rico and isolated in 1955, whereas the BB02 *B. glabrata* strain (genome reference strain [9]) originated from Belo Horizonte, Brazil, and was collected as a field isolate in 2002. Thus, there may be several sources for the genomic variation we observed between Bge3 cells and *B. glabrata* BB02: (i) natural variation that was present in the 1955 albino strain that remains in wild populations of *B. glabrata* but not in BB02; (ii) laboratory and inbreeding selection of BB02 variants since its collection and initial cultivation in 2002; and (iii) laboratory selection of variants during the establishment and passage of the Bge cell line in different locations. Variation in Bge cells can be leveraged to study variation *B. glabrata* genes as well as laboratory-selected mutations that may confer interesting *in vitro* phenotypes. The availability of a genome sequence and variant set for the Bge cell line will allow for more precise design and implementation of experiments incorporating these cells, and it could potentially enable development of novel genetic techniques not previously applicable to this system. For instance, transgenesis of Bge cells has been rarely performed, typically with low transfection efficiencies and transient expression levels [58–60]. However, the availability of the genome sequence could significantly improve the efficacy of Bge cell transgenic approaches by enabling the discovery of strong endogenous promoters. Clearly, such advances make the Bge cell system an attractive *in vitro* model for functional studies of molluscan cell and molecular biology broadly, and specifically the underlying molecular regulation of snail-parasite interactions. Furthermore, with the innovation of gene drives as a potential vector control strategy for mosquitoes [61], there have been suggestions that similar approaches should also be developed in the snails that serve as intermediate hosts for human trematodes and other parasites [11, 62]. Genome editing with CRISPR-Cas9 has not yet been established in *B. glabrata*, and the Bge cell line could first be used to work out the intricacies of double-stranded break repair mechanisms like non-homologous end-joining and homology directed-repair. Finally, all variants called here are hosted by VectorBase [63] and can be readily assessed using its genome browser. Higher confident variant calling can be carried out using integrated consensus calls from multiple algorithms [64], but few variant calling tools are optimized for highly fragmented draft genomes (the *B. glabrata* BB02 reference has 331,401 scaffolds). Thus, as demonstrated here, we recommend the validation of variants by Sanger sequencing before embarking on extensive experimentation with genes that contain them. Nonetheless, natural variation existing between *B. glabrata* BB02, wild populations, and Bge cells will be an important feature to study, and the presented genome and variant calling pipeline will make these studies feasible.

## Methods

### Bge maintenance

The Bge cell line used in this study (Bge3) was purchased from ATCC in the early 1990’s and represents a subculture of the original cell line established by Hansen et al. [15]. Bge3 cells have since been maintained in the laboratories of T. P. Yoshino at the University of Wisconsin-Madison and of M. G. Castillo at New Mexico State University. Cells are routinely cultured at 26 °C under atmospheric conditions in 75 cm^3^ flasks containing complete Bge medium [cBge: 22% Schneider *Drosophila* medium (Lonza, Basel, Switzerland), 0.13% galactose, 0.45% lactalbumin hydrolysate (Sigma-Aldrich, St. Louis, MO, USA), and 10% fetal bovine serum (FBS, Atlanta Biologicals, Flowery Branch, GA, USA) supplemented with penicillin and streptomycin (100 units/ml and 100 μg/ml, respectively, Hyclone, Logan, Utah, USA)]. Typically, Bge3 cells are passaged when confluence reaches 90–100% and re-seeded at various densities (30–60%) in fresh cBge medium according to experimental need, usually once every 7–15 days.

### Genomic DNA isolation

Genomic DNA (gDNA) for sequencing was extracted from Bge3 cells following a 2% CTAB extraction protocol [65]. Bge3 cells from two flasks at 80–100% confluence were used for gDNA extraction. cBge media was removed and cells were washed and pelleted in sterile snail phosphate-buffered saline (sPBS; [66]) by centrifugation at 100× *g* for 3 min at 4 °C. After removal of sPBS, 600 μl of CTAB solution [2% w/v CTAB (Sigma-Aldrich), 1.4 M sodium chloride, 20 mM EDTA, 100 mM Tris-HCl, pH 8] containing 0.2% β-mercaptoethanol (Sigma-Aldrich) and supplemented with proteinase K (0.1 mg/ml, Roche, Basel, Switzerland) was added to the pelleted cells and incubated at 60 °C for 30 min with regular inversion. Following incubation, an equal volume of chloroform:isoamyl alcohol (24:1, Sigma-Aldrich) was added, and the sample was centrifuged at 23 °C for 5 min at 16,000× *g*. To precipitate the gDNA, an equal volume of isopropanol was added to the isolated aqueous phase and centrifuged as above. After removing the isopropanol, the precipitated gDNA was incubated at 23 °C in 1 ml of 10 mM ammonium acetate (Sigma-Aldrich) in 75% ethanol for 15 min. The sample was centrifuged again and the pelleted gDNA was washed with 75% ethanol, spun down, and allowed to dry before dissolving in water. Finally, gDNA was treated with RNase A, cleaned following the manufacturers’ guidelines, and precipitated using a standard phenol:chloroform protocol. Briefly, 10 mg/ml of RNase A (Promega, Madison, WI, USA) was added to the gDNA sample and incubated at 37 °C for 1 h, followed by addition of an equal volume of phenol:chloroform:isoamyl alcohol (25:24:1, Sigma-Aldrich). After centrifugation at 16,000× *g* for 5 min at room temperature, the upper phase was isolated and 5 M ammonium acetate in 100% ethanol (Thermo Fisher Scientific, Waltham, MA, USA) was used to precipitate RNA-free gDNA overnight at -20 °C. Precipitated gDNA was collected by centrifugation at 13,000× *g* for 15 min at 4 °C, washed in 70% ethanol, dried, and suspended in nuclease-free water. The sample was quantified using a Nanodrop spectrophotometer (ND1000; ThermoFisher Scientific) and subjected to agarose gel electrophoresis (1.2% gel; Invitrogen, Carlsbad, CA, USA) for quality control (data not shown).

### Illumina sequencing and analyses

Library preparation and Illumina sequencing was performed at the National Center for Genome Resources (Santa Fe, New Mexico). The DNA library was prepared using the Bge3 cell line gDNA and the Illumina TruSeq DNA kit (FC-121-1001). Library quality was confirmed on an Agilent Bioanalyzer and sequenced on a single lane of an Illumina HiSeq 2500 with 2×100 read lengths.

### Alignment and coverage measurement

The *in silico* pipeline is diagrammed in Additional file 13, and the scripts used for analysis and plotting can be found at www.github.com/zamanianlab/BgeVars. Paired-end reads in FASTQ format were trimmed and filtered with Trimmomatic v0.36 [67]. Scaffold sequences from version 1 of the *B. glabrata* BB02 reference genome [9], which had been softmasked with RepeatMasker [68], DUST [69], and TRF [70], were downloaded from VectorBase [63], and the Bge3 cell reads were aligned to this reference with BWA v0.7.15 [71] and sorted with samtools v1.4.1 [24]. Genome coverage was measured with BedTools v2.26.0 [72] using the -bga flag. The average coverage for each scaffold was calculated using R. To compare coverage between the Bge3 and *B. glabrata* genome assemblies, paired-end reads from the snail genome (SRR024007-8, 17-28, 31-41) [9] were realigned to their reference and coverage was calculated as above. Linkage groups (LGs) for scaffolds were assigned according to the mapping done in *B. glabrata* strain 13-16-R1 [10]. Scaffold coverage calculations were normalized by multiplying each scaffold’s coverage by its length and dividing this product by the sum of all the scaffolds’ coverages multiplied by their lengths. Scaffolds with length >10,000 were used for plotting. Analysis, filtering, and plotting were performed with the *tidyverse* package [73] in custom R [74] scripts.

### Variant identification and filtration

Variants were identified with bcftools v1.4.1 [24], using the -m and -A flags to allow for multiallelic calling and reporting of alternate alleles that do not fit within the forced diploid genotype. For calculating variant allele frequencies, raw variants were refined by retaining single-nucleotide variants (SNVs), filtering for biallelism (sites that were either homozygous within Bge3 but differed from the reference or were heterozygous in Bge3 but shared one allele with the reference), and removing low quality variants [75]. Reference and alternative allele counts were extracted by querying the DP4 INFO tag from the final filtered variant call format (VCF) file. For molecular karyotyping, reference and alternate frequencies were calculated as ratios to total observation count (sum of DP4) at each SNV position. Frequencies < 0.125 and > 0.875 were removed so that, theoretically, allele frequencies corresponding to octosomy and below would be retained. For plotting, only SNVs with read depth of less than 10,000 but greater than 50 were used. SNV analysis, filtering and plotting were performed with vcftools v1.1.15 [76], vcflib GitHub commit 9e116cb [77], and the *tidyverse* package [73] in custom R [74] scripts.

### Variant annotation

SnpEff was used to predict and annotate variant effects [28]. Version 1.6 of the *B. glabrata* gene predictions and annotations was downloaded from VectorBase. High-impact variants were analyzed for Gene Ontology (GO) [78, 79] and for enrichment by employing Fisher’s exact test with the R package *topGO* [80].

### Confirmation of selected variants through PCR and Sanger sequencing

Bge3 genomic DNA was extracted following the DNAzol reagent (Invitrogen, Carlsbad, CA, USA) protocol. The gDNA was treated with RNase A, cleaned, and precipitated following the manufacturer and standard phenol:chloroform protocols as earlier described. gDNA concentration was measured using a BioPhotometer plus (Eppendorf, Hamburg, Germany), diluted to a final concentration of 100 ng/μl, and run on a 1.2% agarose gel for quality control (data not shown). DNA primers were designed using the Integrated DNA Technologies (IDT) PrimerQuest Tool. Primer pairs were designed to flank the predicted SNV for each region of interest. Primers sequences used for PCR confirmation and Sanger sequencing are listed in Additional file 12. Bge3 RNA-free gDNA was used to amplify each of the variant-containing regions by routine PCR using the Q5 Hot Start High-Fidelity 2X Master Mix (New England BioLabs, Ipswich, MA) and following the manufacturer's instructions. Primers for the ribosomal protein subunit-19 (RPS19) were used as positive control. PCR products were analyzed by gel electrophoresis, and sample concentrations were measured using a BioPhotometer plus before being sent for sequencing using the SimpleSeq Premixed DNA Sequencing Service (Eurofins Genomics, Louisville, Kentucky, USA). The resulting Bge3 sequences were aligned with the snail genome using the National Center for Biotechnology Information (NCBI) nucleotide alignment BLAST tool, and SNVs were identified and analyzed.

### Karyotyping

Chromosome preparation was performed as previously described [23], with few modifications. Colcemid (10 ug/ml, Thermo Fisher Scientific) was added to a flask of 70–80% confluent Bge3 cells to a final concentration of 0.13 μg/ml and incubated for 3 h at 26 ^o^C. Cells were freed from the flask, transferred to a 15 ml sterile conical tube, and centrifuged at 100× *g* for 8 min. The supernatant was aspirated and gently replaced with 10 ml hypotonic solution (2 parts 0.0075 M potassium chloride solution to 1 part 0.8% sodium citrate solution), followed by incubation for 20 min at 26 °C. Next, 0.5 ml of 3:1 (MeOH:HOAc) was added and tubes were inverted to mix. Tubes were incubated at room temperature for 5 min, after which they were centrifuged. The resulting supernatant was aspirated, 4 ml of 3:1 fixative was added, and tubes were incubated for 30 min at room temperature. This 30-min fixation step was repeated, and cells were resuspended in 2:1 fixative. Cells were dropped onto slides at appropriate densities and baked for 50 min at 90 °C in a drying chamber (Model C-30, Percival Scientific, Perry, IA, USA).

For Giemsa/Trypsin/Leishman (GTL) staining, baked slides were immersed in trypsin EDTA (5.0 ml 10X trypsin EDTA, 45 ml Hanks solution, 0.5 ml 0.4 M disodium phosphate) for 20 s, rinsed with tap water, immersed in Leishman’s stain working solution consisting of 1 part Leishman’s stain stock solution (1.0 g Leishman’s stain in 500 ml methanol) to 3 parts Gurr buffer (1 Gurr buffer tablet in 1 l water) for 1.75 min, and rinsed again in tap water. Slides were dried with compressed air and mounted. Cells at metaphase were imaged at 100× with an Olympus BX4 microscope. Metaphase chromosomes of individual cells were captured with a Leica Biosystems digital camera, and the Leica Cytovision software was used to generate karyograms. Using previous Bge karyotypes as guides [23, 30] matching chromosomes from at least 20 cells were paired and grouped by size and centromere location.

## Additional files

Additional file 1: Pipeline of the computational workflow. (PDF 127 kb)

Additional file 2: Average read depth coverage (RDC) for a subset of *B. glabrata* BB02 genomic reads re-mapped to the reference. (PDF 344 kb)

Additional file 3: Log2 transformed RDC. **a** Log2 transformed version of Fig. 1. **b** Log_2_-transformed version of Additional file 2. (PDF 683 kb)

Additional file 4: Cell 1 karyotype. (PDF 137 kb)

Additional file 5: Cell 2 karyotype. (PDF 123 kb)

Additional file 6: Cell 3 karyotype. (PDF 128 kb)

Additional file 7: Cell 4 karyotype. (PDF 124 kb)

Additional file 8: Cell 5 karyotype. (PDF 122 kb)

Additional file 9: Cell 6 karyotype. (PDF 119 kb)

Additional file 10: Cell 7 karyotype. (PDF 122 kb)

Additional file 11: A possibly tetraploid Bge3 cell. (PDF 706 kb)

Additional file 12: Selected proteins of interest with high-impact mutations. Selected proteins of interest predicted to have high-impact mutations were grouped by putative functions according to current NCBI or UniProt annotations. BGLB / NCBI accession numbers and their predicted identities are provided. For each entry, the exact location of the mutation in the scaffold is indicated, followed by the nucleotide observed in the snail (reference allele) and the predicted mutated Bge nucleotide (alternative allele). DP4 scores, indicating the numbers of forward and reverse high-quality reads of the reference and the alternate, respectively, as well as the predicted high-impact effect are also presented. Eighty proteins were selected and the fragment containing the mutation submitted to PCR amplification. Each mutation was then verified by Sanger sequencing. The resulting PCR-amplification success or failure, as well as the resulting confirmation of mutation is reported. Remaining proteins are described as unchecked. (XLSX 28 kb)

Additional file 13: Primers used for PCR and Sanger Sequencing. List of forward and reverse primers used to amplify selected fragments of Bge3 genomic DNA containing predicted high-impact mutations. Sequences were later Sanger sequenced and analyzed to confirm mutations. Amplicon sizes and annealing temperatures used for each amplification reaction are listed. (XLSX 13 kb)

## Abbreviations

Bge: *Biomphalaria glabrata* embryonic cell line
CCN: Chromosome copy number
gDNA: Genomic DNA
GTL: Giemsa/Trypsin/Leishman
LG: Linkage group
RADseq: Restriction site-associated DNA markers
RDC: Read depth coverage
RNAi: RNA interference
SNV: Single-nucleotide variant
sPBS: Snail phosphate-buffered saline
