# Correction to: Sequence and structural variation in the genome of the Biomphalaria glabrata embryonic (Bge) cell line

**DOI:** 10.1186/s13071-018-3135-7

**Published:** 2018-10-29

**Authors:** Nicolas J. Wheeler, Nathalie Dinguirard, Joshua Marquez, Adrian Gonzalez, Mostafa Zamanian, Timothy P. Yoshino, Maria G. Castillo

**Affiliations:** 1 Department of Pathobiological Sciences, School of Veterinary Medicine, University of Wisconsin, Madison, WI USA; 2 Department of Biology, New Mexico State University, Las Cruces, NM USA

## Correction

Following publication of the original article [1], the authors reported an error in figure 1:

The incorrect **Figure 1** published is:

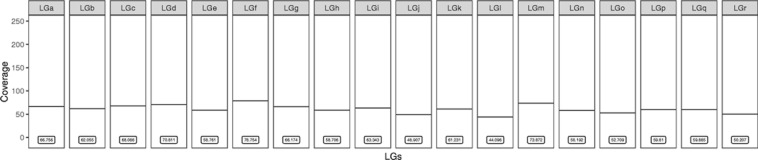



The correct **Figure 1** is:


