# Supplementary figures and images for: Sequence and structural variation in the genome of the Biomphalaria glabrata embryonic (Bge) cell line
Source: Parasit Vectors. 2018 Sep 4;11:496. doi: 10.1186/s13071-018-3059-2 (PMC6122571; doi:10.1186/s13071-018-3059-2)

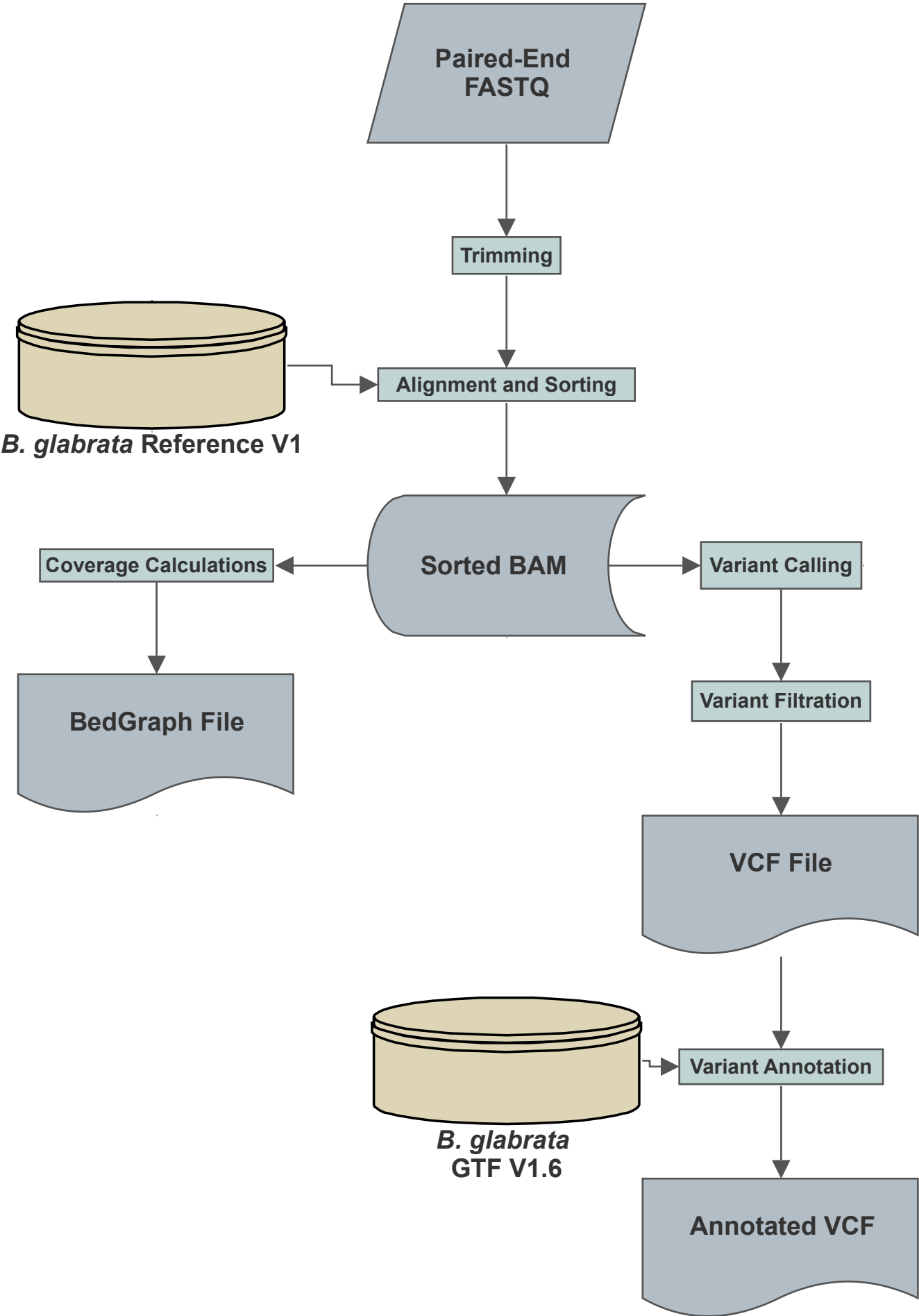

Supplement: Supplementary file 1 — Pipeline of the computational workflow. (PDF 127 kb) [file 13071_2018_3059_MOESM1_ESM.pdf]

Coverage

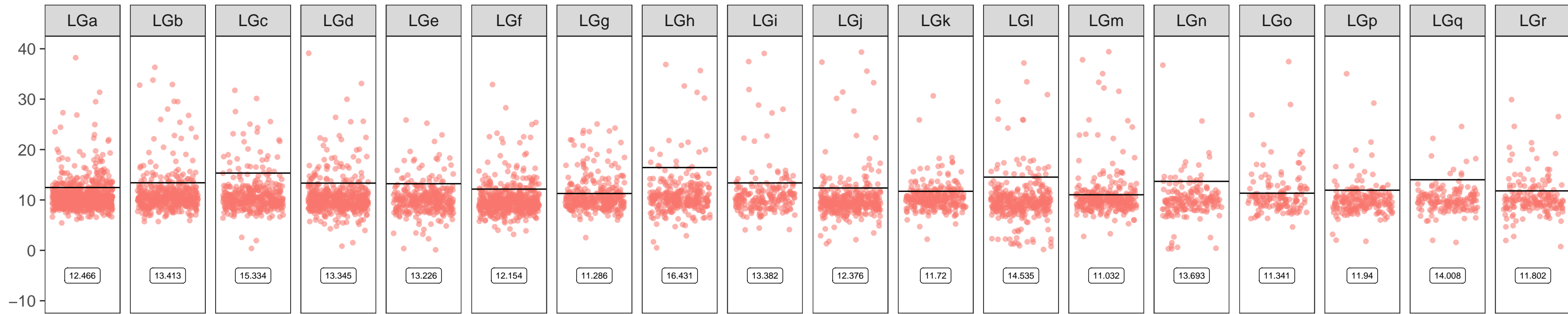

LGs

Supplement: Supplementary file 2 — Average read depth coverage (RDC) for a subset of B. glabrata BB02 genomic reads re-mapped to the reference. (PDF 344 kb) [file 13071_2018_3059_MOESM2_ESM.pdf]

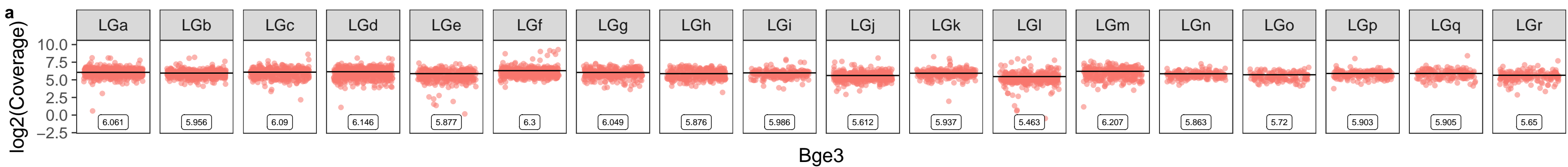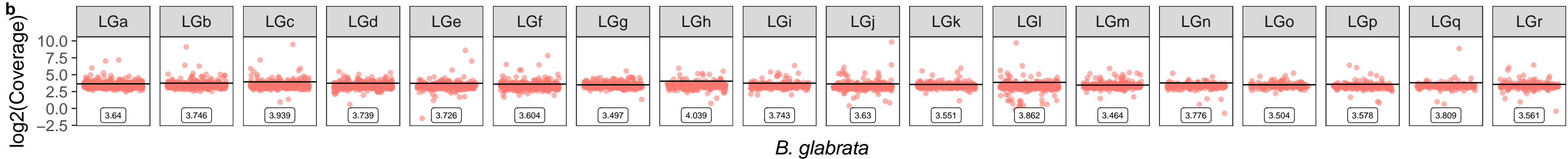

Supplement: Supplementary file 3 — Log2 transformed RDC. a Log2 transformed version of Fig. 1. b Log2-transformed version of Additional file 2. (PDF 683 kb) [file 13071_2018_3059_MOESM3_ESM.pdf]

# Cell 1

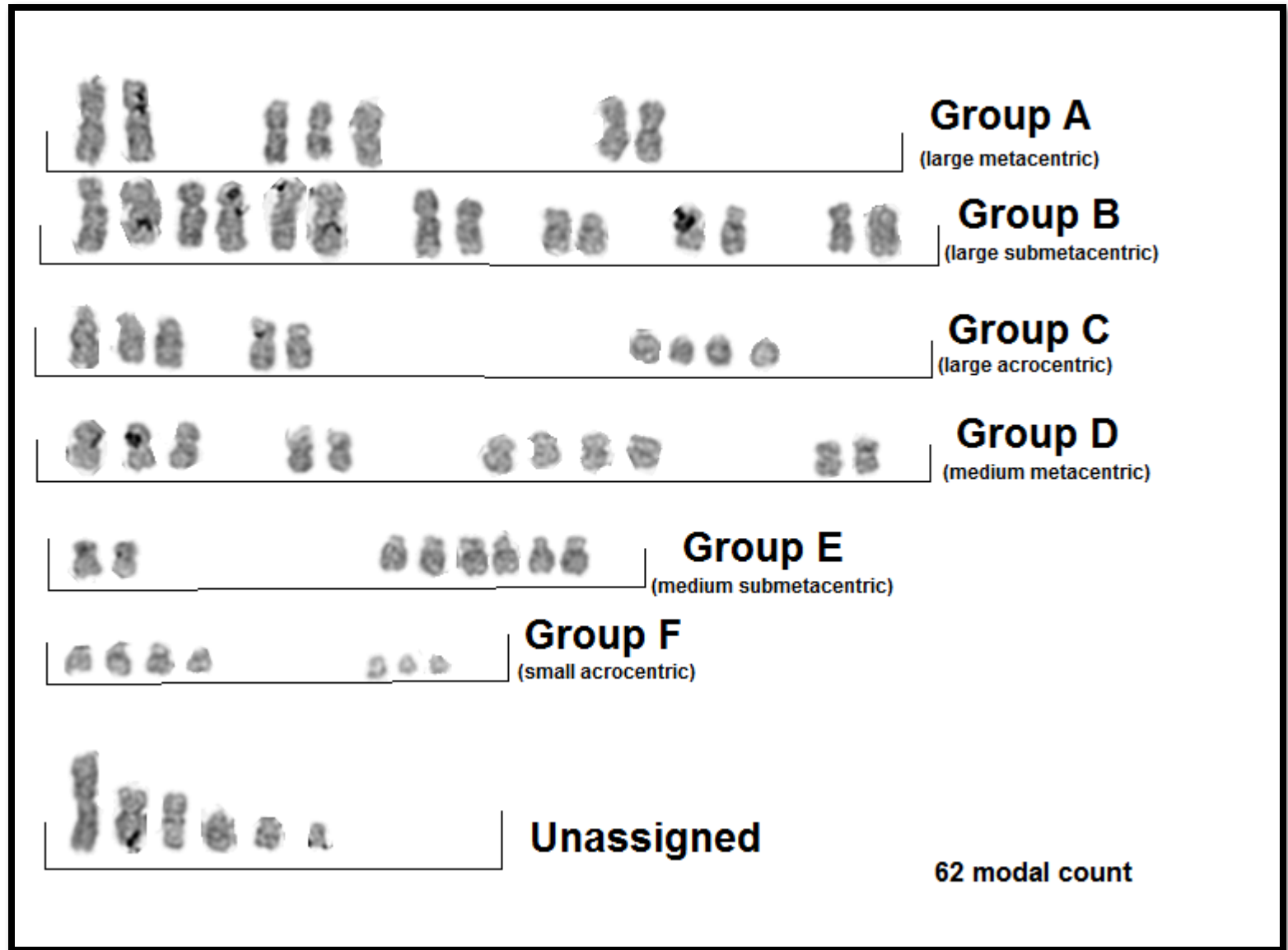

Supplement: Supplementary file 4 — Cell 1 karyotype. (PDF 137 kb) [file 13071_2018_3059_MOESM4_ESM.pdf]

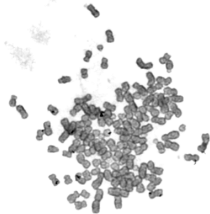

Supplement: Supplementary file 11 — A possibly tetraploid Bge3 cell. (PDF 706 kb) [file 13071_2018_3059_MOESM11_ESM.pdf]
