# Supplementary material for: Sequence and structural variation in the genome of the Biomphalaria glabrata embryonic (Bge) cell line
Source: Parasit Vectors. 2018 Sep 4;11:496. doi: 10.1186/s13071-018-3059-2 (PMC6122571; doi:10.1186/s13071-018-3059-2)
Supplement: Supplementary file 8 — Cell 5 karyotype. (PDF 122 kb) [file 13071_2018_3059_MOESM8_ESM.pdf]

Cell 5

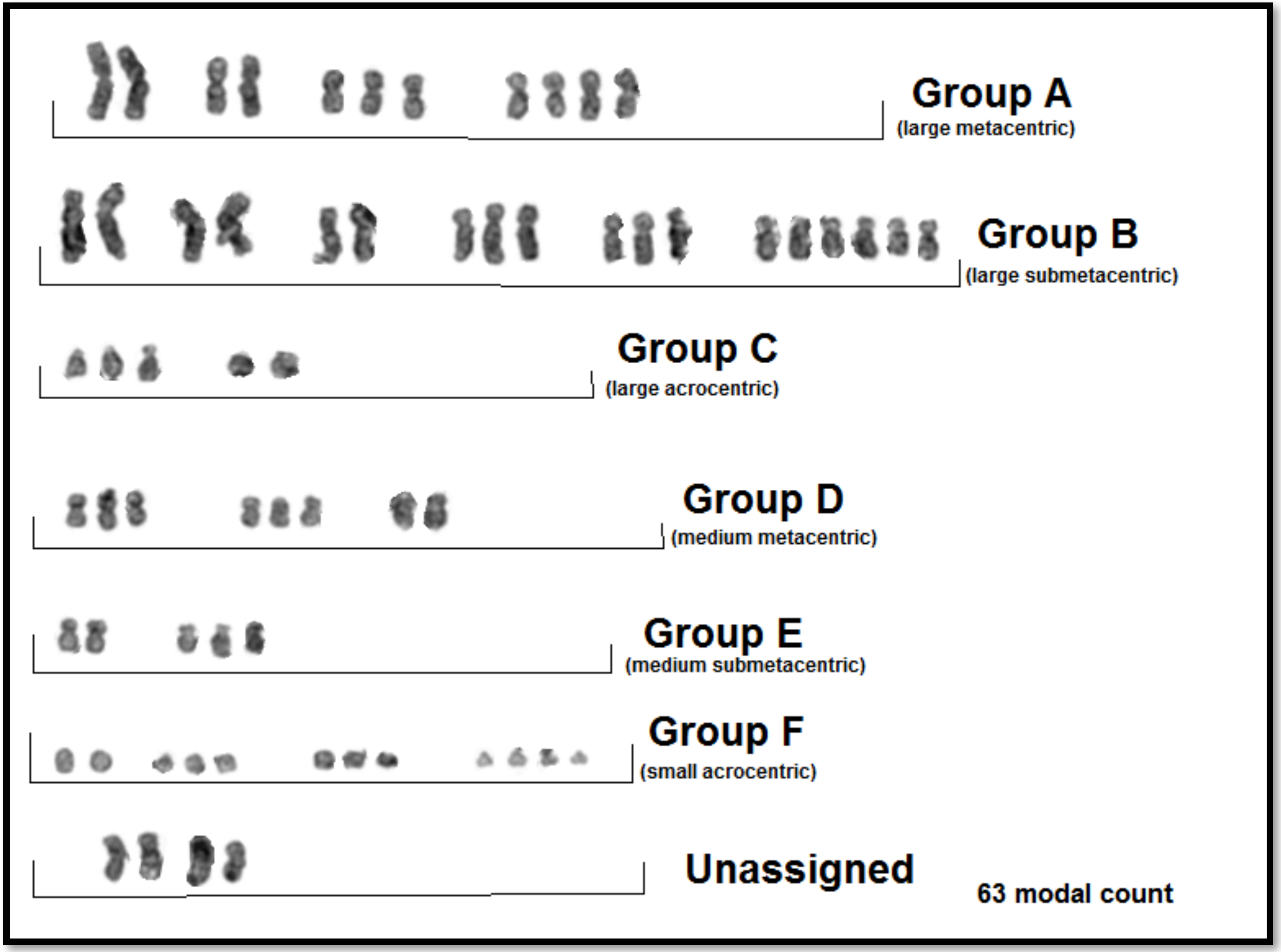
Group A
(large metacentric)
Group B
(large submetacentric)
Group C
(large acrocentric)
Group D
(medium metacentric)
Group E
(medium submetacentric)
Group F
(small acrocentric)
Unassigned
63 modal count
